# Protective effect of mangiferin on myocardial ischemia-reperfusion injury in streptozotocin-induced diabetic rats: role of AGE-RAGE/MAPK pathways

**DOI:** 10.1038/srep42027

**Published:** 2017-02-09

**Authors:** Kapil Suchal, Salma Malik, Sana Irfan Khan, Rajiv Kumar Malhotra, Sameer N. Goyal, Jagriti Bhatia, Santosh Kumari, Shreesh Ojha, Dharamvir Singh Arya

**Affiliations:** 1 Department of Pharmacology, Cardiovascular Research Laboratory, All India Institute of Medical Sciences, New Delhi-110029, India; 2 Department of Pharmacology, R.C. Patel Institute of Pharmaceutical Education and Research, Shirpur, Maharashtra-425405, India; 3 Indian Agricultural Research Institute, New Delhi 110012, India; 4 Department of Pharmacology and Therapeutics, College of Medicine and Health Sciences, United Arab Emirates University, Al Ain, Abu Dhabi 17666, United Arab Emirates

## Abstract

Hyperglycemia induced advanced glycation end products-receptor for advanced glycation end products (AGE-RAGE) activation is thought to involve in the development of cardiovascular disease in diabetics. Activation of AGE-RAGE axis results in the oxidative stress and inflammation. Mangiferin is found in the bark of mango tree and is known to treat diseases owing to its various biological activities. Thus, this study was designed to evaluate the effect of mangiferin in ischemia-reperfusion (IR) induced myocardial injury in diabetic rats. A single injection of STZ (70 mg/kg; i.p.) was injected to male albino Wistar rats to induce diabetes. After confirmation of diabetes, rats were administered vehicle (2 ml/kg; i.p.) and mangiferin (40 mg/kg; i.p.) for 28 days. On 28^th^ day, left anterior descending coronary artery was ligated for 45 min and then reperfused for 60 min. Mangiferin treatment significantly improved cardiac function, restored antioxidant status, reduced inflammation, apoptosis and maintained myocardial architecture. Furthermore, mangiferin significantly inhibited the activation of AGE-RAGE axis, c-Jun N-terminal kinase (JNK) and p38 and increased the expression of extracellular regulated kinase 1/2 (ERK1/2) in the myocardium. Thus, mangiferin attenuated IR injury in diabetic rats by modulation of AGE-RAGE/MAPK pathways which further prevented oxidative stress, inflammation and apoptosis in the myocardium.

Globally, the incidence of cardiovascular disease in diabetics is far greater as compared to the non diabetic population^1^. Amongst the cardiovascular diseases, ischemic heart disease (IHD) serves the most common cause of death in diabetic conditions. Furthermore, despite the available therapy options, fatality in patients with IHD remains high; the letter being more pronounced in diabetics^2^. The restoration of blood supply is the mainstay of treatment in IHD. During therapy of the ischemic myocardium, the revascularization of occluded blood vessel causes rapid reperfusion which has been shown to have deleterious effects such as inflammation and generation of reactive oxygen species (ROS)^3^. It is well established that reperfusion accelerates the development of necrosis and histological changes observed after 30–60 minutes of ischemia-reperfusion (IR) is very well comparable to the degree of what normally occurs after 24 hours of permanent coronary occlusion^4^. Furthermore, myocardial IR causes an inflammatory response which leads to damage of the viable tissue surrounding the infarct, the proposed mechanism of the latter being accelerated apoptosis^5^. The detrimental effect of myocardial reperfusion has been shown to be more pronounced in diabetic patients^6^.

The persistent hyperglycemia observed in the setting of uncontrolled diabetes mellitus leads to the formation of advanced glycation end products (AGE)^7^. The process of glycation is one of the major mechanisms which contribute to the development as well as progression of various complications of diabetes including cardiomyopathy, nephropathy, retinopathy and neuropathy^8^. The effect of AGE in diabetes may be either direct and/or indirect. The direct mechanism by which glycation interferes with the cell function include denaturation of the target protein as well as decline in its function, accumulation of AGEs in tissues leading to organ malfunction, and generation of oxidative stress^9^. The indirect mechanism is a receptor mediated mechanism involving the receptor for advanced glycation end products (RAGE)^10^. It has been demonstrated that RAGE expression is increased in various conditions such as inflammation, myocardial ischemia-reperfusion, as well as in the micro- and macrovascular complications of diabetes^11^,^12^. Furthermore, it has been shown that increased RAGE augments the synthesis of ROS and increased oxidative stress upregulate the RAGE, thus leading to a positive feedback loop^13^,^14^,^15^. One of the consequences of oxidative stress is the phosphorylation of the major signal transduction cascade molecule mitogen activated protein kinase (MAPK) which has several effects, one of them being the activation of nuclear transcription factors including NF-κB which is involved in the genetic regulation of various cytokines and chemokines^16^. Since, AGE-RAGE induced oxidative stress is involved in the pathogenesis of myocardial IR injury in diabetics; its inhibition can serve as an important means of preventing and alleviating IR injury.

Over the years, substantial amount of natural products have been investigated for ameliorating hyperglycemia in diabetics. Fruits, vegetables, wine and tea constitute a significant source of flavonoids, xanthanoids or polyphenolic compounds. Mangiferin, which is a natural C-glucosyl xanthone and polyhydroxy polyphenol compound, is found in bark of mango tree (*Mangifera indica*)^17^. Several previous studies have shown that mangiferin has antioxidant^18^, anti-inflammatory^19^, anti-apoptotic^20^ and anti-diabetic effects^21^. Mangiferin has shown to possess anti-AGE properties as mangiferin is reported to significantly ameliorate diabetic cardiomyopathy by preventing AGE-RAGE production^22^. Keeping the recent reports in mind, the present study was designed as a mechanistic approach to determine the effect of mangiferin on the myocardial IR injury in diabetic rats and further to investigate the mechanism involve in its cardioprotection.

## Material and Methods

### Antibodies and chemicals

Antibodies against Extracellular regulated kinase 1/2 (ERK1/2), phospho (p)-ERK1/2, c-Jun N-terminal kinase (JNK), p-JNK, NF-κBp65, Caspase-3 and β-actin were obtained from Cell Signaling Technology, USA. Primary antibodies for Bcl-2, Bax, CD-45 and p38 were procured from Abcam, UK. Phospho p38 (p-p38) and RAGE primary antibodies were from Santa Cruz, USA. Horseradish peroxidase linked goat-anti rabbit and goat-anti mouse secondary antibodies were obtained from Merck Genei, India. Enzyme Linked Immunosorbent Assay (ELISA) kits for Advanced glycation end products (AGEs), Creatine kinase-MB (CK-MB), Lactate dehydrogenase (LDH), Rat tumor necrosis factor-α (TNF-α) and Rat interleukin-6 (IL-6) were purchased from Korain Biotech Co. Ltd., China, Spinreact, Spain, Logotech Private Limited, India, Diaclone Tepnel Company, UK and RayBiotech, Inc., Norcross, GA respectively. One touch Ultra 2 Blood Glucose Meter for measuring blood glucose level was procured from LifeScan, Inc. Milpitas, CA. Terminal deoxynucleotide transferase dUTP nick end labeling (TUNEL) assay kit was obtained from Biovision Inc. California. All other chemicals were purchased from Sigma Chemicals (St. Louis, MO. USA) and were of analytical grade.

### Test drug

Mangiferin and Streptozotocin (STZ) were supplied by Sigma Chemicals (St. Louis, MO. USA) respectively. For administering to the rats, mangiferin was dissolved in 0.5% dimethyl sulfoxide (DMSO).

### Experimental preparation

Adult male albino Wistar rats (150–200 g) obtained from central animal house facility of All India Institute of Medical Sciences, New Delhi, were housed in departmental animal house in controlled temperature (25 ± 2 °C), relative humidity (60 ± 5%) with 12 h light/dark cycle. All the rats were acclimatized one week prior to the experiment. The experimental protocol was carried out in accordance to Indian National Science Academy guidelines for use and care of experimental animals in research and approved by the Institutional animal ethics committee (813/IAEC/14). During the entire experimental period, rats had free access to tap water and food pellets (Ashirwad Industries Ltd, Chandigarh, India) *ad libitum*.

### Induction of diabetes

Diabetes was induced by injecting a single intraperitoneal injection of STZ (70 mg/kg, 0.1 M cold citrate buffer, pH 4.5) to the overnight fasted rats. Diabetes in animals was confirmed 3 days post STZ injection using Glucose Meter. Rats with fasting blood glucose level more than 250 mg/dl were considered as diabetic and included in the further experiment.

### Drug administration and animal model of myocardial IR injury

Diabetic rats (n = 34) were randomly divided into following 3 groups. Group 1 (Diabetic control; n = 10): Diabetic rats were administered 0.5% DMSO (2 ml/kg/day; i.p) for 28 days and on 28^th^ day, rats underwent entire surgical procedure but occlusion of left anterior descending (LAD) coronary was not performed. Group 2 (Diabetes + IR; n = 12): Diabetic rats were administered 0.5% DMSO (2 ml/kg/day; i.p) for 28 days and on the 28^th^ day, myocardial IR injury was induced by ligation of LAD coronary artery for 45 min followed by reperfusion for 60 min. Group 3 (Mangiferin 40 + Diabetes + IR; n = 12): Diabetic rats were administered mangiferin (40 mg/kg/day; i.p) for 28 days and on the 28^th^ day, myocardial IR injury was induced by ligation of LAD coronary artery for 45 min followed by reperfusion for 60 min.

### Determination of hemodynamic parameters

The myocardial IR injury was performed according to the previous procedure (Suchal *et al*., ref. 23). Briefly, diabetic rats were anaesthetized with pentobarbitone sodium. Blood was withdrawn from tail vein and fasting blood glucose levels in all rats were measured using one touch Ultra 2 Blood Glucose Meter. After assessment of blood glucose level, LAD coronary artery was ligated for 45 min and thereafter, ligation was removed and reperfusion was done for 60 min. Hemodynamic parameters i.e. systolic arterial pressure (SAP), mean arterial pressure (MAP), diastolic arterial pressure (DAP), maximal rate of change of left ventricular pressure (±LVdP/dt) and left ventricular end diastolic pressure (LVEDP) were measured using BIOPAC system software BSL 4.0 MP36. Blood was drawn *via* cardiac puncture, centrifuged at 4000 rpm and stored in −20 °C till analysis. After measurement of hemodynamic parameters, rats were sacrificed with an overdose of pentobarbitone sodium (150 mg/kg; i.p.). Then, hearts were excised, washed with ice-chilled normal saline and stored in liquid nitrogen for biochemical and western blot analysis, in 10% neutral buffered formalin for histopathological, immunohistochemistry (IHC) and TUNEL assay and in Karnovsky’s fixative for ultrastructural evaluation.

### Measurement of cardiac lipid peroxidation and antioxidants

Heart tissue was removed from liquid nitrogen and homogenised with 0.1 M phosphate buffer (pH 7.4). Half part of this homogenate was used for the estimation of malondialdehyde (MDA) level^24^, a marker of lipid peroxidation, and reduced glutathione (GSH) content^25^. Other part of the homogenate was centrifuged at 5000 rpm at 4 °C. The clear supernatant thus obtained was used for the measurement of superoxide dismutase (SOD)^26^ and catalase (CAT)^27^ enzyme activities and protein content^28^.

### Assessment of cardiac enzyme activities

Activities of cardiac enzymes i.e. CK-MB and LDH were assessed in the serum using commercially available kits.

### Estimation of TNF-α, IL-6 and AGEs levels

The levels of serum TNF-α, IL-6 and AGEs were estimated using ELISA kits following manufacturer’s instructions.

### Morphological evaluation of cardiac tissue

For histopathological evaluation, heart tissues fixed in 10% neutral buffered formalin were embedded in paraffin to make tissue blocks. The blocks were then cut into 5 μm thick histologic section with microtome (Leica RM 2125, Germany). The sections were then stained with hematoxylin and eosin (H&E). At least three hearts from each group were examined under light microscope (Dewinter technologies, Italy) for any pathological changes. The findings were reported as (−) no change; (+) focal change; (++) patchy change; (+++) confluent change.

For ultrastructural evaluation heart tissues stored in Karnovsky’s fixative were washed with ice-chilled phosphate buffer (0.1 M, pH 7.4) and then fixed with 1% osmium tetroxide at 4 °C. Following this, tissues were embedded in araldite CY212 and tissue blocks were made. Ultrathin sections of 70–80 nm thickness were cut using an ultramicrotome (Ultracut E, Reichert, Austria) and stained with uranyl acetate and lead acetate. The sections were then visualized under transmission electron microscope (Morgagni268D, FeiCo., The Netherlands).

### Immunohistochemistry (IHC)

Heart tissues were sectioned using microtome and sections were placed on slides. Then slides were passed through xylene to graded series of ethanol to deparaffinized and rehydrate tissue sections. After this, antigen in tissue section was retrieved by heating in a microwave at 95 °C for 10 min in citrate buffer (10 mM; pH 6.0). Following this, sections were blocked with 30% hydrogen peroxide (H_2_O_2_) in methanol for 30 min and with normal goat serum for 2 h to reduce endogenous peroxidase and non specific binding respectively. Then, the sections were incubated with primary antibodies against Bcl-2, Bax, Caspase-3 and CD-45 for 48 h. For detection of primary antibodies, sections were incubated with secondary antibodies for 2 h. Colorimetric reaction was initiated with addition of DAB. The sections were stained with hematoxylin, mounted with DPX and examined under light microscope at 40X magnification (Dewinter technologies, Italy).

### TUNEL assay

Heart sections (5 μm) thickness were obtained using microtome. The sections were deparaffinized in xylene and rehydrated in graded concentration of ethanol. Following this, sections were then incubated with Proteinase K and with 30% H_2_O_2_ to enhance tissue permeability and to diminish any endogenous peroxidase activity respectively. Then sections were incubated with complete labeling reaction buffer and antibody solution. Colorimetric reaction was visualized using 3,3-diaminobenzidine (DAB). Sections were stained with hematoxylin and visualized under light microscope (Dewinter technologies, Italy).

Histopathology, ultrastructural, IHC and TUNEL slides were evaluated by an expert pathologist blinded to the treatment protocol.

### Western blot analysis

Heart tissues were removed from liquid nitrogen and homogenised in RIPA buffer (150 mM NaCl, 10% Triton X-100, 0.5% Sodium deoxycholate, 0.1% Sodium dodecyl sulphate, 50 mM Tris base) containing protease inhibitor (Sigma Aldrich, USA). Homogenate was centrifuged at 12000 rpm for 20 min at 4 °C and supernatant was used for estimation of protein content. Sample equal to (40 μg) protein concentration was separated on sodium dodecyl sulphate polyacrylamide gel electrophoresis (SDS-PAGE). Protein was then transferred to nitrocellulose membrane. Membrane was subsequently blocked with 3% bovine serum albumin (BSA) for 2 h at room temperature. Following this, membrane was washed three times with tris buffer saline containing 0.5% tween-20 (TBS-T). Membrane was then incubated with primary antibodies (ERK1/2, p-ERK1/2, JNK, p-JNK, p38, p-p38, NF-κBp65, RAGE and β-actin) overnight at 4 °C. After incubation with primary antibodies, membrane was again washed 3 times with TBS-T and incubated with secondary antibodies for 2 h at room temperature. The bands were developed with an enhanced chemiluminescence (ECL) (Thermofischer Scientific Inc. USA) kit and band intensity was analyzed by image j software.

### Statistical analysis

Statistical analysis was performed by one way analysis of variance (ANOVA) followed by Tukey-Kramer multiple comparison post-hoc test with Graph Pad InStat software. Data have been expressed as mean ± S.E.M. A value of P < 0.05 was considered significant.

## Results

### Mortality rate

During the entire study period, a total of 14.7% mortality was observed due to bleeding or improper ligation of LAD coronary artery and diabetes.

### Body weight changes

In all experimental groups, body weight was measured at 1st, 7th, 14th, 21st and 28th day of the treatment protocol. In all the groups, there was significant variation in body weight as compared to their respective baseline values (P < 0.05). However, no significant change in body weight was observed among the experimental groups (Table 1).

### Blood glucose level

Fasting blood glucose level in all experimental groups was measured at 1st, 7th, 14th, 21st and 28th day. No significant difference in blood glucose level was observed in all the groups as compared to their baseline values. However, mangiferin treatment to diabetic rats significantly (P < 0.05) reduced the blood glucose level as compared to diabetes + IR and diabetic control groups (Table 2).

### Mangiferin improved hemodynamic and ventricular functions

**Figure 1:**
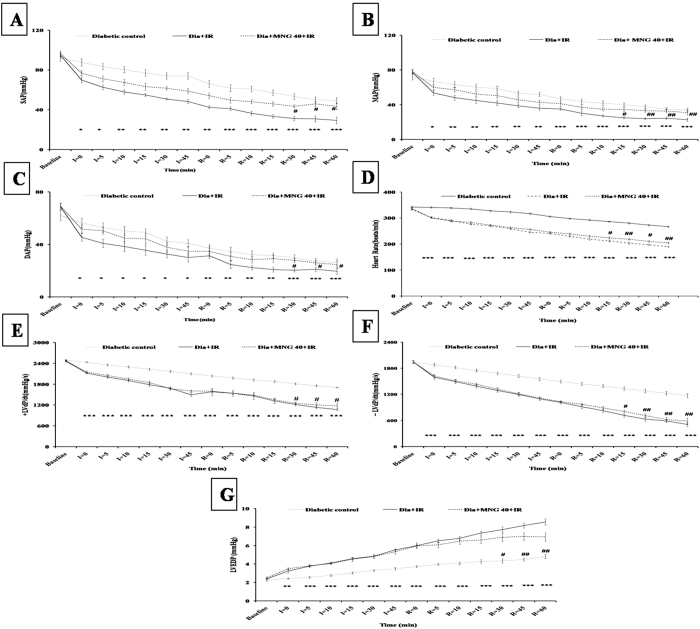
Effect of mangiferin on hemodynamic and ventricular functions in various experimental groups. (**A**) SAP; (**B**) MAP; (**C**) DAP; (**D**) HR; (**E**) Maximal positive rate of the left ventricular pressure (+LVdP/dtmax); (**F**) Maximal negative rate of the left ventricular pressure (−LVdP/dtmax); (**G**) LVEDP. Dia + IR: diabetes + ischemia-reperfusion; Dia + MNG 40 + IR: diabetes + mangiferin 40 mg/kg + ischemia-reperfusion. Data are expressed as mean ± S.E.M.; n = 6 per group. *P < 0.05, **P < 0.01, ***P < 0.001 vs. Diabetic control; ^**#**^P < 0.05, ^**##**^P < 0.01 vs. Dia + IR.

As shown in Fig. 1, diabetes + IR group exhibited significant (P < 0.001) impairment in hemodynamic parameters as demonstrated by reduction in arterial pressure and HR. In addition to hemodynamic impairment, there was significant ventricular dysfunction i.e. decreased rate of contraction (+LVdP/dt) and relaxation (−LVdP/dt) along with increase in pre-load (LVEDP) in comparison to diabetic control group. Contrary to this, mangiferin administration to diabetic rats significantly improved the hemodynamic (SAP, MAP, DAP and HR) and ventricular functions (±LVdP/dt and LVEDP) as compared to diabetes + IR rats.

### Mangiferin normalized antioxidant status and cardiac enzymes levels

As anticipated, IR insult to the diabetic rats caused significant depletion of antioxidants such as GSH (P < 0.001), SOD (P < 0.05) and CAT (P < 0.01). Similarly, there was increase in the level of MDA (P < 0.001), a marker of lipid peroxidation, in diabetic-IR rats as compared to diabetic control. Furthermore, IR injury disrupted the membrane and caused release of necroenzymes from the myocardium as evident from increased serum level of CK-MB (P < 0.001) and LDH (P < 0.001) in diabetes + IR group. Intriguingly, mangiferin treatment for 28 days significantly bolstered the antioxidant status, reduced MDA level and prevented release of cardiac injury marker after IR injury in diabetic rats (Table 3).

### Mangiferin preserved morphological alterations

**Figure 2:**
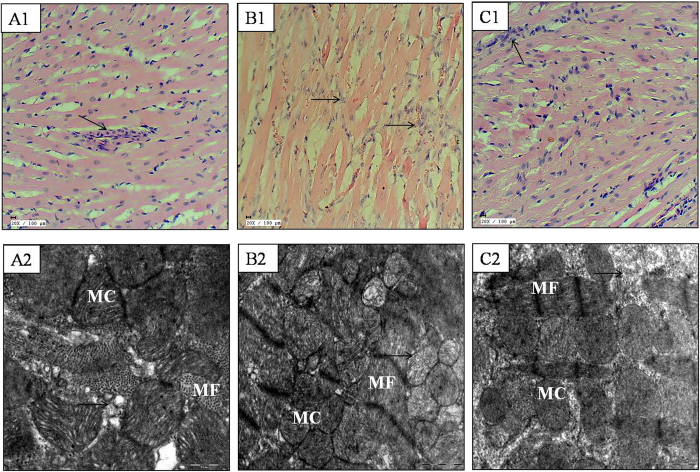
Effect of mangiferin on histological (20X; scale bar: 100 μm; n = 3) and ultrastructural changes (scale bar: 1 μm; n = 3) in various experimental groups. (**A1**,**A2**) diabetic control; (**B1**,**B2**) diabetes + ischemia-reperfusion; (**C1**,**C2**) diabetes + mangiferin 40 mg/kg + ischemia-reperfusion; MF: myofibrils; MC: mitochondria. (→): indicates myocyte damage.

The histopathological sections of rat hearts in the diabetes + IR group showed marked myonecrosis, edema and inflammation along with higher histological score. In mangiferin treatment group, the myocardium appeared normal with less necrosis, edema and inflammation (Fig. 2). These changes are graded and summarised in Table 4.

In line with the histopathological results, the ultrastructural evaluation of myocardium of diabetic-IR rats revealed extensive myofibrillar degeneration, swelling and irregular mitochondria with disorganized cristae. On the other hand, in the mangiferin treatment group, the myofibres had intact bands and normal mitochondria with well preserved cristae (Fig. 2).

### Mangiferin attenuated myocardial apoptosis

**Figure 3:**
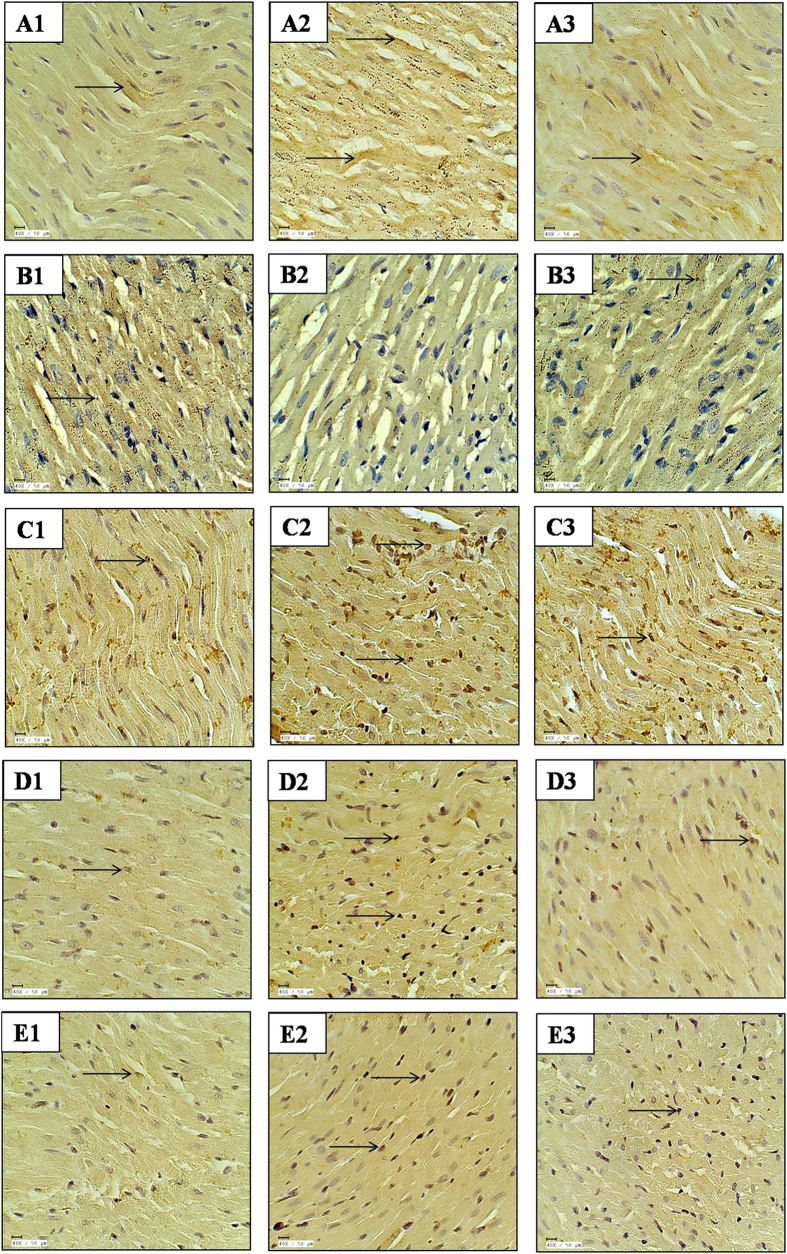
Effect of mangiferin on immunohistochemistry and TUNEL assay in various experimental groups. (**A1**–**A3**) Bax immunohistochemistry; (**B1**–**B3**) Bcl-2 immunohistochemistry; (**C1**–**C3**) CD-45 immunohistochemistry; (**D1**–**D3**): Caspase-3 immunohistochemistry; (**E1**–**E3**): TUNEL positivity; (**A1**–**E1**) diabetic control; (**A2**–**E2**) diabetes + ischemia-reperfusion; (**A3**–**E3**) diabetes + mangiferin 40 mg/kg/day + ischemia-reperfusion; n = 3 per group; 40X; scale bar 50 μm; (→): positive stain.

IHC and TUNEL assay were performed to evaluate the effect of mangiferin on myocardial apoptosis induced by IR injury in diabetic rats. In the diabetes + IR rat hearts, there was increased expression of pro-apoptotic proteins (Bax and Caspase-3) and decreased expression of anti-apoptotic protein Bcl-2. In addition, there was increased number of TUNEL positive cell in diabetes + IR group in comparison to diabetic control group. The results showed that the treatment with mangiferin remarkably increased the Bcl-2 and decreased Bax and Caspase-3 expression in the myocardium. Furthermore, less number of TUNEL positive cells were present in the mangiferin treated group (Fig. 3).

### Mangiferin inhibited inflammation

**Figure 4:**
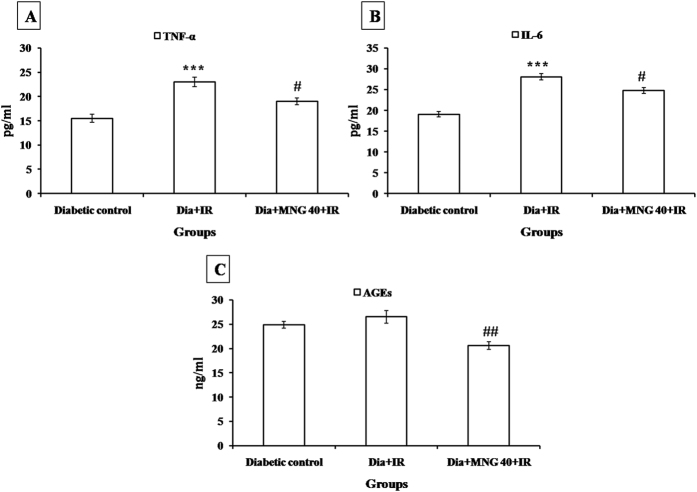
Effect of mangiferin on serum (**A**) TNF-α; (**B**) IL-6; (**C**) AGEs level in various experimental groups. TNF-α: tumor necrosis factor-alpha; IL-6: interleukin-6; AGEs: advanced glycation end products. Dia + IR: diabetes + ischemia-reperfusion; Dia + MNG 40 + IR: diabetes + mangiferin 40 mg/kg + ischemia-reperfusion. All values are expressed as mean ± S.E.M.; n = 6 per group. ***P < 0.001 vs. Diabetic control; ^**#**^P < 0.05, ^**##**^P < 0.01 vs. Dia + IR.

**Figure 5:**
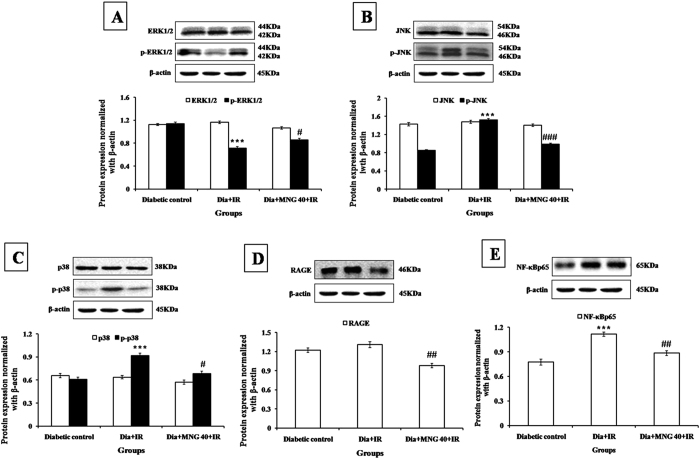
Effect of mangiferin on protein expressions in various experimental groups. (**A**) ERK1/2, p-ERK1/2; (**B**) JNK, p-JNK; (**C**) p38, p-p38; (**D**) RAGE; (**E**) NF-κBp65. Dia + IR: diabetes + ischemia-reperfusion; Dia + MNG 40 + IR: diabetes + mangiferin 40 mg/kg + ischemia-reperfusion. Protein expressions are normalized with β-actin. All the values are expressed as mean ± S.E.M.; n = 3 per group. ***P < 0.001 vs. Diabetic control; ^**#**^P < 0.05; ^**##**^P < 0.01; ^**###**^P < 0.001 vs. Dia + IR.

In diabetes + IR group, there was increased number of CD-45 positive cells indicating the presence of inflammatory cells in the myocardium. Furthermore, there was increased expression of NF-κBp65, a transcription factor and serum TNF-α and IL-6 levels in diabetes + IR group as compared to diabetic control group (P < 0.001). Interestingly, mangiferin treatment significantly reduced inflammation as there was decreased level of inflammatory markers as compared to diabetes + IR rats (Figs 3, 4 and 5).

### Mangiferin attenuated activation of AGE-RAGE

There was increased expression of AGE-RAGE in the diabetic groups. Administration of mangiferin to diabetic rats significantly (P < 0.01) attenuated the formation of AGE and subsequently decreased the expression of AGE-RAGE in comparison to diabetes + IR group (Figs 4 and 5).

### Mangiferin modulated the MAPK phosphorylation

Since, mangiferin strengthened the antioxidant system, attenuated myocardial apoptosis and inflammation, we further explored whether it acts through modulation of MAPK pathway as this signaling pathway is activated by reactive oxygen species. Our western blot data demonstrated that there was increased phosphorylation of stress activated protein kinases such as p38 and JNK and decreased phosphorylation of pro-survival kinase i.e. ERK1/2 in diabetic-IR group as compared to diabetic control group. Treatment with mangiferin significantly normalized this pathway as there was increased ERK1/2 and decreased p38 and JNK expression in the myocardium (Fig. 5).

## Discussion

In the present study, we demonstrated that mangiferin improved cardiac function, inhibited oxidative stress, inflammation and apoptosis in IR challenged diabetic myocardium. The mechanism associated with this protection involves modulation of AGE-RGE/MAPK signaling pathways.

It has been shown that AGEs production increased in the hyperglycemic state and its accumulation is responsible for worsening of IR injury in diabetics^29^,^30^. AGE acts through binding to its receptor RAGE which is upregulated in diseased conditions such as inflammation, vascular disease, neurodegenerative disorder and cancer^31^. Previous reports have documented that administration of AGEs to the animals resulted in increased MDA, level and treatment with anti-RAGE antibodies or antioxidants diminished the deleterious effect of AGEs^32^. Furthermore, in another study, binding of AGE to RAGE augmented synthesis of highly reactive oxygen species (ROS) and caused oxidative stress which further worsened diabetes and its progression^33^. AGE-RAGE activation has shown to increase the production of ROS which further facilitated the generation of mitochondrial superoxide anion in diabetic mice^34^. Similar to previous study, we found significantly increased level of MDA and depletion of antioxidants in the myocardium. Increased MDA disrupted the membrane integrity thus there was increased release of cardiac injury markers i.e. CK-MB and LDH in the serum. Interestingly, pre-treatment with mangiferin for 28 days reduced hyperglycemia, prevented AGE-RAGE activation and attenuated oxidative stress in the diabetic myocardium. Our findings are supported by a study in which mangiferin has improved the activity of cardiac and renal antioxidant enzyme activity in STZ-induced diabetic rats^35^. Furthermore, Liu and colleagues showed that mangiferin remarkably ameliorated diabetic nephropathy in rats by inhibiting the AGE-RAGE axis induced oxidative stress damage^36^. Mangiferin also significantly alleviated diabetic cardiomyopathy by preventing the release of inflammatory cytokines, and inhibiting ROS accumulation, AGE-RAGE production, and NF-κB nuclear translocation^22^. Thus, AGE-RAGE mediated oxidative stress suppression is the likely mechanism by which mangiferin improved cardiac function.

Previous studies have reported that binding of AGE to its cell surface receptor RAGE resulted in NF-κB mediated release of pro-inflammatory cytokines^37^,^38^. NF-κB is a transcription factor, which is present in the inactive state in the cells. Upon activation, it translocates into the nucleus, where it promotes the transcription of many genes including those encoding for inflammatory cytokines such as TNF-α, IL-6 and adhesion molecules^39^. Our results supported this by demonstrating increased serum TNF-α/IL-6 levels and NF-κBp65 expressions in IR insulted diabetic myocardium. Contrary to this, treatment with mangiferin decreased the level of inflammatory markers. In an *in vivo* study, Pal and colleagues demonstrated that mangiferin suppressed inflammatory lesion in diabetic nephropathy by reducing TNF-α and IL-6 levels along with reduced expression of IKK and subsequent inhibition of NF-κB pathway activation^40^. These molecular changes were further backed up by inflammatory and necrotic changes in the tissue’s histology. There was significant edema, myonecrosis and infiltration of inflammatory cells in diabetes + IR rats. Mangiferin decreased necrosis as well as inflammation and near-normal morphological structure was observed in the pre-treated rats. Ultrastructural findings also supported the molecular results as evidenced by distruption of cristae, swollen and irregular mitochondria with nuclear and chromatin condensation in the myocardium of diabetes + IR rats. However, mangiferin treatment maintained the structural integrity; as there was mild mitochondrial swelling without any chromatin condensation. Thus, findings from the current study propose that mangiferin can serve as a novel therapeutic strategy for attenuation of inflammation associated with myocardial IR injury in the setting of diabetes.

Furthermore, an association has been found in increased AGE-RAGE axis and MAPK pathway^41^,^42^. MAPK’s belong to the family of protein kinases and comprise of extracellular signal-regulated kinase 1 and 2 (ERK1/2), c-Jun N-terminal kinase (JNK) and p38^43^. It has been demonstrated that ERK is involved in the regulation of cell proliferation as it is activated by growth factors^44^. The protective role of ERK during an ischemic injury has been demonstrated in a study which showed that ERK may be an important factor for the cell to survive^45^. Inversely, the activation of JNK and p38 MAP kinases primarily leads to apoptosis^46^. Ku and colleagues have shown that diallyl trisulfide protected against high glucose induced cardiomyocyte apoptosis by reducing oxidative stress and by inhibiting JNK pathway^47^. Furthermore, in another study, oligonol administration attenuated renal damage *via* inhibition of AGE-RAGE/JNK medicated inflammation and apoptosis^48^. Similarly, in this present study, there was increased expression of JNK, p38, Bax and decreased expression of ERK1/2 and Bcl-2 in the diabetes + IR group while an opposite effect was seen in the mangiferin treatment group. The present results are in accordance with the previous findings which demonstrate that anti-apoptotic property of mangiferin is responsible for its cardioprotective effect^23^,^49^.

In conclusion, results from our study established that, treatment with mangiferin favourably modulated AGE-RAGE/MAPK pathways which further prevented oxidative stress, inflammation and apoptosis in the IR-induced myocardial injury in diabetic rats.

## Additional Information

**How to cite this article:** Suchal, K. *et al*. Protective effect of mangiferin on myocardial ischemia-reperfusion injury in streptozotocin-induced diabetic rats: role of AGE-RAGE/MAPK pathways. *Sci. Rep.* **7**, 42027; doi: 10.1038/srep42027 (2017).

**Publisher's note:** Springer Nature remains neutral with regard to jurisdictional claims in published maps and institutional affiliations.

## Figures and Tables

**Table 1:** Body weight changes in various experimental groups.

Groups	1^st^ day	7^th^ day	14^th^ day	21^st^ day	28^th^ day
Diabetic control	153.58 ± 4.30	148.26 ± 5.86	144.27 ± 4.76	141.43 ± 2.18	134.04 ± 3.57*
Dia + IR	150.55 ± 5.47	146.09 ± 4.34	141.70 ± 4.41	138.06 ± 3.66	132.32 ± 2.81*
Dia + MNG 40 + IR	161.31 ± 4.49	155.02 ± 4.69	152.10 ± 3.03	148.07 ± 2.82	141.51 ± 4.41*

Dia + IR: diabetes + ischemia-reperfusion; Dia + MNG 40 + IR: diabetes + mangiferin 40 mg/kg + ischemia-reperfusion. All values are expressed as mean ± S.E.M.; n = 6 per group. *P < 0.05 vs. baseline value in respective groups.

**Table 2:** Blood glucose level in various experimental groups.

Groups	1^st^ day	7^th^ day	14^th^ day	21^st^ day	28^th^ day
Diabetic control	497 ± 13.23	495.17 ± 11.01	500.17 ± 10.74	501.83 ± 16.31	503.5 ± 10.99
Dia + IR	512.18 ± 15.40	507.05 ± 17.15	510.02 ± 18.56	503.13 ± 14.86	505.31 ± 11.88
Dia + MNG 40 + IR	483.19 ± 23.03	474.95 ± 23.25	468.74 ± 19.27	463.72 ± 12.82	457.87 ± 12.88*

Dia + IR: diabetes + ischemia-reperfusion; Dia + MNG 40 + IR: diabetes + mangiferin 40 mg/kg + ischemia-reperfusion. All values are expressed as mean ± S.E.M.; n = 6 per group. *P < 0.05 vs. Dia + IR and diabetic control groups.

**Table 3:** Oxidative stress and cardiac injury markers in various experimental groups.

Groups	MDA (nmole/g tissue)	GSH (μmole/g tissue)	SOD (U/mg protein)	CAT (U/mg protein)	LDH (U/L)	CK-MB (U/L)
Diabetic control	67.19 ± 3.11	1.02 ± 0.07	4.35 ± 0.48	5.96 ± 0.56	505.94 ± 21.99	422.66 ± 23.72
Dia + IR	103.41 ± 4.05***	0.50 ± 0.05***	2.10 ± 0.51*	3.22 ± 0.31**	764.98 ± 28.40***	662.6 ± 25.59***
Dia + MNG 40 + IR	87.54 ± 3.38^**#**^	0.77 ± 0.05^**#**^	4.03 ± 0.49^**#**^	4.97 ± 0.42^**#**^	650.29 ± 23.05^**#**^	561.06 ± 15.45^**##**^

MDA: malondialdehyde; GSH: reduced glutathione; SOD: superoxide dismutase; CAT: catalase; LDH: lactate dehydrogenase; CK-MB: creatine kinase-MB isoenzyme. Dia + IR: diabetes + ischemia-reperfusion; Dia + MNG 40 + IR: diabetes + mangiferin 40 mg/kg + ischemia-reperfusion. All values are expressed as mean ± S.E.M.; n = 6 per group. *P < 0.05, **P < 0.01, ***P < 0.001 vs. Diabetic control; ^**#**^P < 0.05, ^**##**^P < 0.01 vs. Dia + IR.

**Table 4:** Histopathological assessment of heart tissues from various experimental groups.

Groups	Necrosis	Edema	Inflammation
Diabetic control	−	−	+
Dia + IR	+++	+++	+++
Dia + MNG 40 + IR	+	−	++

The changes are ranked as: (−): no change; (+): focal change; (++): patchy change; (+++): confluent change. Dia + IR: diabetes + ischemia-reperfusion; Dia + MNG 40 + IR: diabetes + mangiferin 40 mg/kg + ischemia-reperfusion; n = 3 per group.
